# A Prospective Study of Comparison of Left Ventricular Volumes and Function by 2D and 3D Transthoracic Echocardiography in Patients With Left Ventricular Systolic Dysfunction

**DOI:** 10.7759/cureus.115412

**Published:** 2026-08-29

**Authors:** Sunanda Mohanty, Anupam Jena, Binayananda Padhee, Pradeep Sareddy, Nelson Ghosh, Subasis Mishra, Suryakant Jena, Panchanan Sahoo, Hrudananda Mishra

**Affiliations:** 1 Cardiology, Kalinga Institute of Medical Sciences, Bhubaneswar, IND

**Keywords:** heart failure, left‑ventricular ejection fraction, regional wall‑motion abnormalities, three‑dimensional echocardiography, two‑dimensional echocardiography, ventricular volume quantification

## Abstract

Background: Accurate assessment of left ventricular ejection fraction (LVEF) and volumes is pivotal for stratifying heart‑failure severity and guiding device or revascularisation therapy. Two‑dimensional echocardiography (2DE) is ubiquitous, yet geometric assumptions impair precision. Three‑dimensional echocardiography (3DE) offers improved spatial resolution, but its added value in routine practice is under‑reported.

Objectives: The objectives of this study were to compare 2DE‑ and 3DE‑derived LVEF and volumes in heart failure with reduced ejection fraction, quantify reclassification rates (patients whose classification of the severity of LV systolic dysfunction changes between 2DE and 3DE), and identify subgroups benefiting most from 3DE (e.g., patients having moderate LV systolic dysfunction by 2DE who were found to have severe LV systolic dysfunction by 3DE were advised for implantable cardioverter-defibrillator or cardiac resynchronisation therapy).

Methods: This prospective observational study enrolled 104 patients with left ventricular systolic dysfunction (ejection fraction (EF) <50%) at Kalinga Institute of Medical Sciences between March 2020 and September 2022. Two-dimensional (2D) and three-dimensional (3D) transthoracic echocardiography (TTE) were performed using a Philips Epiq™ 7 system with S5-1 and X5-1 transducers, respectively. LV volumes and EF were measured using Simpson's biplane and Teichholz methods for 2DE, and full-volume acquisition for 3DE. Visual EF estimation ("eyeballing") was also recorded. Intra-modality and inter-modality comparisons were analysed using paired t-tests, Pearson correlation coefficients, and Bland-Altman plots.

Results: Three-dimensional datasets were successfully acquired in 102 (98.1%) of 104 patients. The mean age was 56.4±11.5 years, with 72 (69.2%) male patients. EF measured by 3DE (34.8±11.9%) was significantly lower compared to that measured by 2D Teichholz (37±10.4%) and Simpson's method (37.1±12.8%; p<0.001). Eyeballing EF (34.9±10.1%) showed the strongest correlation with 3DE (r=0.701). Strong correlations were observed between 3DE- and 2DE-derived volumes (end-diastolic volume (EDV) r=0.82-0.83; end-systolic volume (ESV) r=0.83-0.86), though Bland-Altman analysis showed wide limits of agreement. 3DE reclassified 17 (43.5%) of 39 patients with moderate LV systolic dysfunction by 2DE into the severe dysfunction category. Regional wall motion abnormality (RWMA) was identified in 69 (67.6%) patients by 3DE versus 39 (38.5%) by 2DE. Correlation between 2DE and 3DE was better in patients with global hypokinesia than in those with RWMA. Image acquisition and analysis averaged 23.1±3.8 minutes.

Conclusions: Adding 3DE to routine 2DE refines quantification of LVEF, ventricular volumes, and regional function, reclassifying nearly half of borderline cases and potentially altering defibrillator or resynchronisation therapy recommendations. Where 3DE is unavailable, expert visual estimation outperforms formula‑based 2DE in approximating 3DE accuracy and risk stratification in daily practice.

## Introduction

Transthoracic echocardiography (TTE) has become integral to modern cardiology practice. Several echocardiographic techniques are available, each with distinct advantages and limitations in evaluating cardiac structure and function. Two-dimensional echocardiography (2DE) is the most commonly used method for quantifying left ventricular (LV) function. Among its techniques, the Teichholz method calculates ejection fraction (EF) using linear dimensions from parasternal long-axis views, assuming a fixed geometric model of the LV. While simple and rapid, it is highly dependent on the assumption of a symmetric LV shape, which may not hold true in conditions like regional wall motion abnormalities (RWMAs) or ventricular remodeling. The Simpson’s biplane method, recommended by major guidelines, utilises apical two- and four-chamber views to compute LV volumes and EF by summing the areas of stacked discs. This method reduces geometric assumptions but is still subject to limitations such as foreshortened views and interobserver variability.

Three-dimensional TTE (3DE) yields measurements of LV and left atrial (LA) volumes that surpass two-dimensional (2D) techniques in both accuracy and reproducibility, primarily because 3DE eliminates reliance on geometric modelling and is less susceptible to apical foreshortening [1,2]. By acquiring full-volume datasets across several cardiac cycles, 3DE permits detailed LV modelling that encompasses global and regional myocardial performance, mechanical dyssynchrony, and wall motion abnormalities. On the strength of these advantages, current imaging guidelines endorse the clinical application of 3DE for LV volume quantification [3,4]. Importantly, contemporary ultrasound platforms now allow both 2D and 3DE datasets to be obtained with a single transducer, thereby streamlining the incorporation of 3DE into everyday practice. Non-contrast 3DE has demonstrated the highest measurement reproducibility for both left ventricular ejection fraction (LVEF) and LV volumes across serial assessments spanning 12 months [5]. Despite this growing body of evidence favouring the diagnostic superiority of 3D echocardiography, its incorporation into routine clinical workflows remains limited. The present study therefore sought to determine whether supplementing standard 2DE with 3DE in patients who have LV systolic dysfunction yields meaningfully different EF and volume measurements, and whether such differences carry implications for therapeutic decision-making.

## Materials and methods

Aims and objectives

The aims of this study were to compare 2DE- and 3DE-derived LVEF and volumes in patients with LV systolic dysfunction (screening EF <50%), and to quantify reclassification of severity grade between 2DE and 3DE. As a pre-specified secondary analysis, we assessed potential reclassification across the 35% EF threshold (<35% vs ≥35%) and identified subgroups in which the two methods differed most (e.g., patients graded as moderate by 2DE who were reclassified as severe by 3DE). Treatment decisions, device implantation, and clinical outcomes were not evaluated.

Methods

Study Design

This prospective observational single-centre study was conducted at the Kalinga Institute of Medical Sciences (KIIT University) between March 2020 and September 2022.

Ethical Considerations

The study was approved by the Institutional Ethics Committee of the Kalinga Institute of Medical Sciences (KIMS), KIIT Deemed-to-be-University, Bhubaneswar (Ref. No.: KIIT/KIMS/IEC/262/2020, dated 17.03.2020). Written informed consent was obtained from each patient.

Study Criteria

Eligible participants were adults aged 18-80 years who had LV systolic dysfunction of either ischaemic or non-ischaemic origin (screening EF below 50%, determined by 2D Simpson's biplane at the index clinical echocardiogram) and were in sinus rhythm on electrocardiography. Exclusion criteria encompassed inadequate echocardiographic windows precluding 3D analysis, translational cardiac artefacts, atrial fibrillation, frequent premature ventricular complexes, haemodynamically significant (moderate or greater) valvular disease, and inability to sustain a brief apnoeic period of 15-20 seconds required for volumetric acquisition. Recruitment was consecutive among eligible patients presenting during the study period: 194 patients were screened, and 90 were excluded for an inadequate acoustic window, atrial fibrillation, frequent ventricular ectopy, haemodynamically significant valvular disease, or inability to sustain the required breath-hold. A small number of enrolled patients had an EF ≥50% on subsequent quantification, consistent with expected screening-to-study variability.

Study Procedures

Three‑dimensional echocardiography imaging was performed on a Philips EPIQ 7 platform equipped with an X5 matrix-array transducer (Philips Healthcare, Andover, MA, USA) while each subject lay in the left lateral recumbent position. Wide-angled acquisition using “full-volume” mode over 4 beats was used during a single breath-hold. Care was taken to include the entire LA and LV cavity within the 3D volume. Imaging settings were optimised for endocardial visualisation. The highest possible frame rate was obtained by minimising imaging depth and sector width. Two‑dimensional echocardiography imaging was done including apical two-, three-, and four-chamber views, from which LVEF was derived by applying the Simpson’s biplane disc-summation algorithm (S5-1 phased-array TTE transducer). Both 3DE and 2DE datasets were analysed with the vendor’s integrated software, which employs semi-automated endocardial border detection supplemented by manual refinement where needed.

Tools and Assessments (Image Analysis)

The system's inbuilt software package (3DQAdv, Philips Healthcare, Andover, MA, USA) was used for offline quantitative analysis of the LV volumes and function. The possibility of stitching artefacts was checked in a transversal plane using the slicing (particularly multislice) function, which was also used to analyse wall motion and distribution of hypertrophy. The software semi-automatically detected and traced the endocardial border of the LV during the cardiac cycle. Manual corrections were done as needed. A cast of the LV was created, allowing comprehensive volumetric analysis of LV function and morphology. Time-volume curves were generated to evaluate segmental LV function and the presence of dyssynchrony. Finally, the segmental contraction patterns were displayed graphically through parametric imaging in a dynamic ‘bulls eye’ display, providing information on segmental volume changes and timings of segmental contraction. RWMAs were determined as per the 17-segment model of the left ventricle laid down by the American Heart Association (AHA) guidelines [6].

RWMAs were classified into severity categories as per American Society of Echocardiography (ASE) recommendations using the following scoring system: (1) normal or hyperkinetic, (2) hypokinetic (reduced thickening), (3) akinetic (absent or negligible thickening, e.g., scar), and (4) dyskinetic (systolic thinning or stretching, e.g., aneurysm). EF was graded by the ASE/European Association of Cardiovascular Imaging (EACVI) 2015 chamber-quantification criteria [4] as normal (≥52%), mildly abnormal (41-51%), moderately abnormal (30-40%), or severely abnormal (<30%) on both 2DE and 3DE; this echocardiographic severity grading is reported separately from clinical heart failure phenotyping. Severity grades were derived directly from the measured EF by applying these boundaries. Any changes in grading of EF or RWMA by 3DE were documented [7].

See Appendix for definitions.

Sample Size Calculation

Assuming a mean difference in LVEF of 3% by 2DE and 3DE with 10% standard deviation, a sample size of 88 was required to achieve 80% power of the study. We enrolled 104 subjects.

Statistical Analysis

Continuous data are reported as mean ± standard deviation (SD) and categorical data as frequencies (%). All inter-method comparisons used pairwise complete data; the two patients without an adequate 3DE dataset were excluded from 3DE comparisons (n = 102; Simpson n = 103), with no imputation. The normality of the paired differences was assessed with the Shapiro-Wilk test. Because most paired differences departed from normality, the Wilcoxon signed-rank test was the primary test of inter-method difference, with the paired-samples t-test reported alongside for comparability; each comparison reports the paired difference, its 95% confidence interval, the test statistic, and the exact two-tailed p-value (significance p < 0.05). Inter-method agreement for end-diastolic volume (EDV), end-systolic volume (ESV), and LVEF was assessed with Pearson correlation and Bland-Altman analysis, reporting bias and 95% limits of agreement, each with 95% confidence intervals; proportional bias was tested by regressing the between-method difference on the mean of the two methods. Simpson's biplane served as the principal 2D comparator with 3DE, and the Teichholz method was analysed secondarily. Agreement of severity grade was summarised by cross-tabulation with unweighted and linear-weighted Cohen's kappa, and agreement for RWMA between 2DE and 3DE was assessed with McNemar's test and Cohen's kappa. Studies were interpreted by two readers with more than five years of echocardiographic experience; readers were blinded to the results of the other modalities, visual (“eyeballing”) EF was recorded before quantitative analysis, and intra- and inter-observer reproducibility were not assessed.

## Results

A total of 104 patients who satisfied the inclusion and exclusion criteria were enrolled after obtaining written informed consent. Three-dimensional datasets were successfully acquired in 102 (98.1%) of 104 patients; the remaining two were excluded from all 3DE comparisons (pairwise complete analysis, no imputation). The baseline demographic and clinical characteristics of the study population are summarised in Table 1.

**Table 1:** Baseline demographic and clinical characteristics (n = 104) Percentages are of the whole cohort (n = 104). Aetiology was assigned from the recorded clinical diagnosis; the six patients whose only recorded diagnosis was a device implant (cardiac resynchronisation therapy defibrillator or permanent pacemaker) are shown as not specified. 3D, three-dimensional echocardiography; NYHA, New York Heart Association; SD, standard deviation.

Characteristic	Value
Age (years), mean ± SD	56.4 ± 11.5
Male sex, n (%)	72 (69.2%)
Ischaemic aetiology, n (%)	48 (46.2%)
Non-ischaemic aetiology, n (%)	50 (48.1%)
Aetiology not specified, n (%)	6 (5.8%)
Diabetes mellitus, n (%)	12 (11.5%)
NYHA class III, n (%)	45 (43.3%)
Successful 3D acquisition, n (%)	102 (98.1%)
Image acquisition and analysis time (min), mean ± SD	23.1 ± 3.8

Table 2 summarises the mean EF and LV volumes measured by various echocardiographic techniques.

**Table 2:** Mean ejection fraction and left ventricular volumes measured by each echocardiographic method Values are mean ± SD of all available measurements for each method. The number of patients differs between methods because Simpson’s biplane volumes could not be obtained in one patient and an interpretable three-dimensional dataset could not be obtained in two; no data were imputed. Volumes are not derived from visual estimation (--). Between-method differences, calculated only in patients measured by both methods (n = 102), are given in Tables 3, 4. EDV, end-diastolic volume; EF, ejection fraction; ESV, end-systolic volume; SD, standard deviation; 2D, two-dimensional echocardiography; 3DE, three-dimensional echocardiography.

Method	n	Mean EF (%)	Mean EDV (ml)	Mean ESV (ml)
2D Teichholz	104	37.0 ± 10.4	176.9 ± 78.5	114.3 ± 65.3
Simpson’s biplane	103	37.1 ± 12.9	146.4 ± 79.9	94.2 ± 63.0
Eyeballing	104	34.9 ± 10.2	--	--
3DE	102	34.9 ± 12.0	125.0 ± 59.2	83.0 ± 46.8

Mean EFs were similar across all techniques (Table 2). In the paired data (n = 102), the mean EF differences between 2DE and 3DE were small: Teichholz-3DE 1.85% (95% CI −0.21 to 3.91; paired-t p = 0.077, Wilcoxon p = 0.161) and Simpson-3DE 2.24% (95% CI 0.06 to 4.42; p = 0.044/0.024), whereas eyeballing 3DE showed no significant difference (−0.25%; 95% CI −1.96 to 1.45; p = 0.77/0.72). Thus, only the Simpson 3DE EF difference reached statistical significance, and its magnitude (about 2 percentage points) is clinically small (Table 3, Table 4). In contrast, LV volumes differed substantially: EDV was larger by 52.4 ml (95% CI 43.7-61.2) with Teichholz and 21.7 ml (12.7-30.7) with Simpson, and ESV by 32.0 ml (24.7-39.3) and 11.4 ml (5.0-17.9), respectively, all p < 0.001 by the paired t-test and p ≤ 0.003 by the Wilcoxon test. Bland-Altman analysis (Figure 1) showed that, although the Pearson correlation between 2DE and 3DE volumes was strong (r = 0.83-0.86), a high correlation does not imply interchangeability, because the limits of agreement were wide for every comparison. For EF, the limits spanned approximately ±17 percentage points even for eyeballing (bias −0.25%, 95% limits −17.3 to 16.8), a range wider than the EF differences used for device therapy thresholds; a near-zero mean bias therefore indicates little systematic difference but not acceptable agreement in individual patients. Proportional bias was present for eyeballing EF (slope −0.21, p = 0.011), Teichholz EF (−0.21, p = 0.044), and all volume comparisons (p < 0.001). For EDV, Teichholz was systematically larger by 52 ml with 95% limits of agreement of −35 to +140 ml, confirming that absolute LV volumes derived from 2D geometric assumptions are not interchangeable with volumetric 3D data.

**Figure 1:**
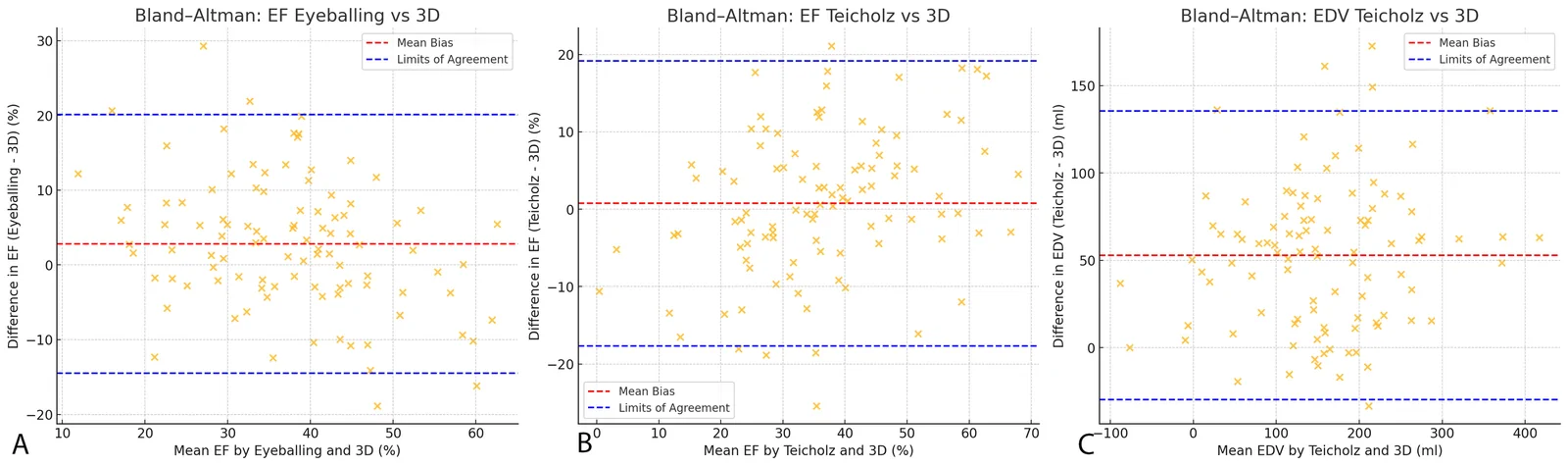
Bland-Altman plots Panel A: LVEF estimated by visual “eyeballing” (2D) versus full-volume 3D echocardiography. The mean bias is −0.25 % (red dashed line), indicating virtually no systematic difference. Ninety five percent limits of agreement (blue dashed lines) range from -17.3% to +16.8%, the narrowest of all comparisons, indicating that, among the 2D approaches, visual assessment had the closest agreement with 3D EF; however, the limits of agreement remain wide and visual EF is not interchangeable with 3D EF. Panel B: LVEF by the Teichholz method (2D) versus 3D echocardiography. The Teichholz algorithm over-estimates EF by 1.9% on average (mean bias, red dashed line). Limits of agreement extend from −18.7% to +22.4%, almost twice the clinically important EF threshold used for device therapy, highlighting the risk of patient misclassification when Teichholz values are substituted for 3D EF. This mean EF difference did not reach statistical significance (p = 0.077); the wide limits of agreement remain the clinically relevant finding. Panel C: EDV by the Teichholz method (2D) versus 3D echocardiography. EDV is systematically larger by 52 ml with 2D Teichholz (mean bias, red dashed line). Limits of agreement are extremely wide (−35 ml to +140 ml), confirming that absolute LV volumes derived from 2D geometric assumptions are not interchangeable with volumetric 3D data. LVEF, left ventricular ejection fraction; EF, ejection fraction; EDV, end-diastolic volume.

**Table 3:** Paired differences in ejection fraction and left ventricular volumes between the two-dimensional methods and 3DE (n = 102) Differences are the two-dimensional value minus the 3DE value; a positive value indicates a higher measurement by the two-dimensional method. p-Values are two-tailed and derived from the paired-samples t-test; the corresponding non-parametric results are given in Table 4. SD of differences is the standard deviation of the within-patient paired differences, not the standard deviation of either measurement. CI, confidence interval; EDV, end-diastolic volume; ESV, end-systolic volume; LVEF, left ventricular ejection fraction; SD, standard deviation; 3DE, three-dimensional echocardiography.

Comparison	Mean difference (95% CI)	SD of differences	p-Value
LVEF (%)			
Teichholz vs 3DE	1.85 (−0.21 to 3.91)	10.47	0.077
Simpson’s vs 3DE	2.24 (0.06 to 4.42)	11.08	0.044
Eyeballing vs 3DE	−0.25 (−1.96 to 1.45)	8.69	0.769
EDV (ml)			
Teichholz vs 3DE	52.4 (43.7 to 61.2)	44.5	<0.001
Simpson’s vs 3DE	21.7 (12.7 to 30.7)	45.9	<0.001
ESV (ml)			
Teichholz vs 3DE	32.0 (24.7 to 39.3)	37.2	<0.001
Simpson’s vs 3DE	11.4 (5.0 to 17.9)	32.8	<0.001

The nonparametric equivalents of the data are also shown in Table 4.

**Table 4:** Wilcoxon signed-rank comparisons of the two-dimensional methods with 3DE (n = 102) Differences are the two-dimensional value minus the 3DE value. Z and p-values are from the two-tailed Wilcoxon signed-rank test using the normal approximation with correction for ties. The interquartile range is the 25th to 75th percentile of the within-patient paired differences. The medians differ appreciably from the corresponding means in Table 3 for the Simpson’s volume comparisons because those paired differences are right-skewed. EDV, end-diastolic volume; ESV, end-systolic volume; IQR, interquartile range; LVEF, left ventricular ejection fraction; 3DE, three-dimensional echocardiography.

Comparison	Median difference (IQR)	Z	p-Value
LVEF (%)			
Teichholz vs 3DE	+1.90 (−5.20 to 7.98)	+1.40	0.161
Simpson’s vs 3DE	+1.85 (−3.40 to 7.70)	+2.26	0.024
Eyeballing vs 3DE	−0.50 (−6.03 to 6.08)	−0.36	0.722
EDV (ml)			
Teichholz vs 3DE	+52.5 (24.9 to 73.0)	+8.16	<0.001
Simpson’s vs 3DE	+10.1 (−5.3 to 35.5)	+4.85	<0.001
ESV (ml)			
Teichholz vs 3DE	+25.9 (12.9 to 46.2)	+7.47	<0.001
Simpson’s vs 3DE	+5.8 (−8.6 to 19.2)	+2.93	0.003

The severity of LV systolic dysfunction was graded from the measured EF by the ASE/EACVI 2015 chamber quantification criteria [4], and the distribution by each method is shown in Table 5. Mild LV systolic dysfunction was recorded in 33 (31.7%) patients by the 2D Teichholz method and in 37 (35.9%) by Simpson’s method. Using eyeballing, 57 (54.8%) patients had moderate LV systolic dysfunction. By 3DE, 37 (36.3%) patients had severe LV systolic dysfunction.

**Table 5:** Severity grading of left ventricular systolic dysfunction by each echocardiographic method Grades were derived from the measured ejection fraction by the ASE/EACVI 2015 chamber quantification criteria: normal ≥52%, mildly abnormal 41-51%, moderately abnormal 30-40%, severely abnormal <30%. Percentages are of the number of patients graded by that method (n column) and sum to 100% across each row; denominators differ because Simpson’s biplane could not be measured in one patient and an interpretable 3DE dataset could not be obtained in two. ASE, American Society of Echocardiography; EACVI, European Association of Cardiovascular Imaging; 2D, two-dimensional echocardiography; 3DE, three-dimensional echocardiography.

Method	n	Normal n (%)	Mild n (%)	Moderate n (%)	Severe n (%)
2D Teichholz	104	5 (4.8%)	33 (31.7%)	42 (40.4%)	24 (23.1%)
Simpson’s biplane	103	12 (11.7%)	37 (35.9%)	27 (26.2%)	27 (26.2%)
Eyeballing	104	3 (2.9%)	19 (18.3%)	57 (54.8%)	25 (24.0%)
3DE	102	10 (9.8%)	23 (22.5%)	32 (31.4%)	37 (36.3%)

Because Simpson’s biplane is the guideline-recommended 2D method, Simpson 3DE was taken as the principal reclassification analysis (Table 6). Of the 102 patients with paired grades, 54 (52.9%) were assigned a different severity category by 3DE than by Simpson (unweighted κ = 0.28, 95% CI 0.16 to 0.39; linear-weighted κ = 0.39, 95% CI 0.14 to 0.63; fair-to-moderate agreement). Across the pre-specified device-relevant threshold, 28 patients (27.5%) crossed 35% EF between Simpson and 3DE (McNemar p = 0.19) and 25 (24.5%) between Teichholz and 3DE (p = 0.23), with no significant directional bias. The Teichholz 3DE cross-tabulation is given in Table 7 as a secondary, descriptive analysis only, because Teichholz is unreliable in remodelled ventricles and in the presence of RWMA.

**Table 6:** Cross-tabulation of severity grade assigned by Simpson’s biplane and by 3DE (principal analysis; n = 102) Values are n (%); percentages are row percentages, that is, the proportion of each Simpson’s grade assigned to each grade by 3DE, and may not sum to exactly 100 owing to rounding. Concordant grades lie on the diagonal: 48 of 102 patients (47.1%) were given the same grade by both methods and 54 (52.9%) were reclassified. Unweighted κ = 0.28 (95% CI 0.16 to 0.39); linear-weighted κ = 0.39 (95% CI 0.14 to 0.63). The two patients without an interpretable 3DE dataset are excluded. CI, confidence interval; 3DE, three-dimensional echocardiography; κ, Cohen’s kappa.

Simpson’s biplane grade	3D echocardiography				Total
	Normal	Mild	Moderate	Severe	
Normal	6 (50.0%)	3 (25.0%)	1 (8.3%)	2 (16.7%)	12 (100%)
Mild	4 (10.8%)	12 (32.4%)	14 (37.8%)	7 (18.9%)	37 (100%)
Moderate	0 (0.0%)	4 (15.4%)	12 (46.2%)	10 (38.5%)	26 (100%)
Severe	0 (0.0%)	4 (14.8%)	5 (18.5%)	18 (66.7%)	27 (100%)
Total	10	23	32	37	102

In the secondary Teichholz 3DE comparison (Table 7), 18 of 42 patients (42.9%) graded as moderate by Teichholz were reclassified as severe by 3DE, and 10 of 32 (31.3%) graded as mild were reclassified as normal; these figures are descriptive and are not used to infer device eligibility.

**Table 7:** Cross-tabulation of severity grade assigned by 2D Teichholz and by 3DE (secondary analysis; n = 102) Values are n (%); percentages are row percentages and may not sum to exactly 100 owing to rounding. Concordant grades lie on the diagonal: 42 of 102 patients (41.2%) were given the same grade by both methods and 60 (58.8%) were reclassified. Unweighted κ = 0.17 (95% CI 0.05 to 0.30); linear-weighted κ = 0.36 (95% CI 0.08 to 0.64). The two patients without an interpretable 3DE dataset are excluded. This comparison is secondary and descriptive, because Teichholz is unreliable in remodelled ventricles and in the presence of regional wall motion abnormality. CI, confidence interval; 2D, two-dimensional echocardiography; 3DE, three-dimensional echocardiography; κ, Cohen’s kappa.

2D Teichholz grade	3D echocardiography				Total
	Normal	Mild	Moderate	Severe	
Normal	0 (0.0%)	2 (50.0%)	0 (0.0%)	2 (50.0%)	4 (100%)
Mild	10 (31.3%)	12 (37.5%)	8 (25.0%)	2 (6.3%)	32 (100%)
Moderate	0 (0.0%)	9 (21.4%)	15 (35.7%)	18 (42.9%)	42 (100%)
Severe	0 (0.0%)	0 (0.0%)	9 (37.5%)	15 (62.5%)	24 (100%)
Total	10	23	32	37	102

Two-dimensional echocardiography identified RWMA in 38 (37.3%) of the 102 patients with an interpretable 3D dataset, and 3DE in 61 (59.8%) (Table 8). Agreement between the methods was moderate (Cohen κ = 0.42), and the difference was significant on paired analysis (McNemar χ² = 15.6, p < 0.001): 27 patients classified as global hypokinesia by 2DE were reclassified as regional by 3DE, versus four in the opposite direction. The 2D-3D correlation was numerically higher in patients with global hypokinesia than in those with RWMA -- for EF, r = 0.56 (95% CI 0.36-0.71; n = 64) versus 0.45 (0.15-0.67; n = 38); for EDV, 0.85 (0.76-0.90) versus 0.67 (0.45-0.82) -- but the confidence intervals overlapped, so the between-subgroup difference was not statistically significant.

**Table 8:** Paired identification of regional wall motion abnormality by 2DE and 3DE (n = 102) Values are numbers of patients. 3DE identified regional wall-motion abnormality in 61 of 102 patients (59.8%) compared with 38 of 102 (37.3%) by 2DE. Twenty-seven patients classified as having global hypokinesia by 2DE were reclassified as regional by 3DE, against four in the opposite direction (McNemar χ² = 15.6, 1 df, p < 0.001). Agreement between the methods was moderate (κ = 0.42, 95% CI 0.27 to 0.57). The two patients without an interpretable 3DE dataset were excluded. CI, confidence interval; RWMA, regional wall motion abnormality; 2DE, two-dimensional echocardiography; 3DE, three-dimensional echocardiography; κ, Cohen’s kappa.

2DE	3DE		Total n (%)
	Global hypokinesia	RWMA	
Global hypokinesia	37	27	64 (62.7%)
RWMA	4	34	38 (37.3%)
Total n (%)	41 (40.2%)	61 (59.8%)	102 (100%)

## Discussion

In this study, mean LVEF was similar between 2DE and 3DE, whereas LV volumes differed substantially, with 2DE yielding larger volumes than 3DE (Table 2). The EF differences were small and, except for Simpson, not statistically significant; the clinically important observation is therefore not a difference in mean EF but the wide limits of agreement and the frequent reclassification of severity category between methods. Using Simpson's biplane as the principal comparator, just over half of the patients (52.9%) were assigned a different severity grade by 3DE, and roughly a quarter crossed the 35% EF threshold in either direction. Baldea et al. examined inter-modality concordance around a 35% LVEF threshold and found that an experienced reader downgraded nearly 15% of patients to an LVEF below 35% while never upgrading any [8]; Hare et al. reported that 3DE had its greatest effect on categorisation among patients whose 2D LVEF fell in the 25-45% range [9]. Our reclassification findings are consistent with these reports; however, because no independent reference standard was used and device eligibility depends on many factors, we did not assess (duration of guideline-directed therapy, symptoms, aetiology, QRS duration and morphology, rhythm, and expected survival), we do not infer device indications from reclassification.

When it comes to LV volumes, the values obtained by 2D and 3D methods in our study were significantly different, but the LV volumes calculated by the two methods were strongly correlated. We found that 2D methods overestimate LV volumes as compared to 3D methods. The lower LV volumes recorded by 3DE relative to 2DE stem from fundamental methodological differences between the two modalities. Because most 2DE algorithms rely on geometric formulae that presuppose a particular LV shape, they are inherently prone to volume and EF misestimation. Hotta et al. in their study evaluated 24 patients with heart failure who were in NYHA functional class III or IV, sinus rhythm with QRS >120 ms, and on an optimised therapy for heart failure undergoing cardiac resynchronisation therapy (CRT) [10]. Their results demonstrated a decrease in both diastolic and systolic LV volumes accompanied by an increase in LVEF following CRT. A strong inter-method correlation was noted for ventricular volumes and LVEF, together with an acceptable correlation for LV mass estimation across both time points. Notably, volume agreement between the two techniques exceeded that observed for LVEF, and Simpson’s-derived LV volumes were consistently larger than those obtained by 3DE, a pattern that mirrors our own observations.

In our subgroup analysis of RWMAs, 38 (37.3%) of the 102 patients with an interpretable 3D dataset had RWMA by 2DE compared with 61 (59.8%) by 3DE, a significant difference on paired testing (McNemar p < 0.001; κ = 0.42). This accords with Hoffmann et al., who found that 3DE detected RWMA better than 2DE across imaging modalities [11]. The 2D-3D correlation for volumes and EF was numerically higher in patients with global hypokinesia than in those with RWMA, but the confidence intervals overlapped and the difference was not statistically significant; we therefore report this pattern descriptively rather than as evidence that any subgroup derives greater benefit. Kotaro Arai et al. reported similar observations [12].

The mean EF by eyeballing did not differ significantly from 3DE (Table 2), and among the 2D approaches, visual estimation had the narrowest limits of agreement with 3DE. This indicates that experienced visual estimation of global LVEF agrees with 3DE at least as closely as formula-based 2D methods do. However, the limits of agreement for visual EF were still wide (approximately ±17 percentage points) and proportional bias was present, so visual estimation is not interchangeable with 3DE and should not be regarded as a substitute for it in individual patients. Visual EF nonetheless remains widely used in routine practice. Lavine and Salacata found that visual and quantitative estimates both correlated with LV global EF [13], and Shahgaldi et al. reported that experienced visual LVEF assessment agreed closely with quantified 3DE-derived EF [14].

Taken together, our findings support using 3DE alongside 2DE in patients with RWMA and moderate LV dysfunction to refine LVEF and volume estimation, while recognising that this study characterises between-method differences and agreement rather than accuracy. Validation studies comparing real-time 3DE with cardiac MRI (CMR) have reported only minor systematic biases in LV volumes and close concordance for EF, which is why we pre-specified 3DE as the reference comparator; however, we did not use CMR and cannot determine which modality is closer to the true value in our patients. CMR remains limited by access and cost, and 2DE by foreshortening and geometric assumptions. Because EF cut-offs of 30% and 35% are used in major guidelines as gatekeeping criteria for implantable cardioverter-defibrillators, between-method disagreement around these thresholds is potentially important; our data quantify the magnitude of that disagreement but do not evaluate device decisions or outcomes.

Our finding that the 2D methods gave slightly higher mean LVEF than 3DE is consistent with the meta-analysis by Dorosz et al., who demonstrated that 2DE systematically overestimates LVEF relative to CMR, whereas 3DE shows closer agreement with CMR-derived values [15]. In a complementary meta-analysis, Shimada and Shiota concluded that real-time 3DE yields LV volume and EF measurements of greater accuracy and consistency than 2DE, with the advantage being most pronounced in patients whose ventricular geometry is distorted [16]. The clinical implication is that echocardiographic severity classification, and hence where a patient falls relative to guideline EF thresholds, depends on which method is used; whether this alters device decisions or clinical outcomes was not evaluated here.

The more frequent detection of RWMA by 3DE observed in our study aligns with the findings by Nesser et al., who reported that 3DE improved wall motion assessment compared with 2DE, particularly in segments that are poorly visualised by conventional imaging planes [17]. Furthermore, Jacobs et al. showed that 3DE-derived strain analysis provided incremental value over 2DE for detecting subtle wall motion abnormalities in patients with coronary artery disease [18].

Our observation that eyeballing correlates well with 3DE-derived EF has practical implications. In resource-limited settings where 3DE may not be available, visual estimation by an experienced operator may serve as a reasonable alternative. However, this approach is operator-dependent and may not be reliable across all levels of expertise. Jenkins et al. emphasised that while visual estimation is widely used, it has inherent limitations in reproducibility compared with quantitative 3DE methods [19].

Limitations of the study

The principal limitation is the absence of CMR or another independent reference standard, so this study can characterise only the differences and agreement between echocardiographic methods, not their accuracy; it cannot establish which modality is closer to true EF or volume, and 3DE was used as a pre-specified reference comparator only based on its prior validation against CMR. Other limitations include the single-centre design and the reliance on operator-dependent acquisition and interpretation; intra- and inter-observer reproducibility were not formally assessed. The severity categories reflect echocardiographic grading and should not be equated with clinical heart failure phenotypes, and reclassification across methods should not be read as a change in device indication.

## Conclusions

In patients with LV systolic dysfunction, 2DE and 3DE yielded similar mean EF but substantially different LV volumes, with wide limits of agreement and frequent reclassification of severity category and RWMA status between methods, particularly by the Teichholz technique. Approximately one-quarter of patients crossed the 35% EF threshold between methods. These findings describe the magnitude of inter-method disagreement and support obtaining 3DE in addition to 2DE when 2D image quality or geometry is limited, especially in patients with RWMA or moderate dysfunction. Because no independent reference standard was used, these differences should not be interpreted as evidence that either modality is more accurate, nor as a basis for device therapy decisions; cost-effectiveness, training requirements, changes in management, and clinical outcomes were not assessed and require dedicated study.

## Appendices

Definitions

End diastole: the frame with the largest LV cavity, at or immediately after mitral valve closure (approximately the onset of the QRS complex).

End systole: the frame with the smallest LV cavity, immediately before mitral valve opening.

Normal and abnormal LV parameters: as per ASE guidelines.
